# No evidence for automatic response activation with target onset in the avatar-compatibility task

**DOI:** 10.3758/s13421-020-01052-2

**Published:** 2020-06-11

**Authors:** C. Böffel, J. Müsseler

**Affiliations:** Work and Cognitive Psychology, RWTH Aachen University, Aachen, Germany

**Keywords:** Avatars, Stimulus response compatibility, Automaticity, Visual perspective taking, Spatial cognition

## Abstract

When people take the perspective of an avatar and perform a stimulus-response compatibility task, they generally show the same compatibility effects that are expected from the avatar’s position instead of their own. In this study, we investigated if these effects are caused by automatic response activation, a concept featured in dual-route models of stimulus-response compatibility. In two experiments we asked 24 participants each to perform a compatibility task from an avatar’s point of view. We introduced a delay between the presentation of the target and the avatar in half of the trials so that the participants had to wait until the avatar appeared to select the correct response. Because the automatic response activation is known to decay quickly, its influence is eliminated in this condition. In contrast to the prediction by the automatic response activation account, we observed a larger compatibility effect in the delayed condition with orthogonal (Experiment 1) and parallel (Experiment 2) stimulus-response pairings. Additionally, distributional analyses of the compatibility effects did not support the automaticity predictions. We conclude that these results call into question the role of automatic response activation for spatial compatibility in general and perspective-based compatibility effects in particular.

**Electronic supplementary material:**

The online version of this article (10.3758/s13421-020-01052-2) contains supplementary material, which is available to authorized users.

## Introduction

Past studies have shown that responses are generally faster for same versus other side reactions when participants have to perform key presses either on the same side (ipsilateral) or on the opposite side (contralateral) of a visual stimulus (e.g., Brebner et al. 1972; Proctor, Yamaguchi, Dutt, & Gonzalez, 2013). An ipsilateral condition would, for example, demand a left key press as an answer to a disc presented on the left side of the screen, while a contralateral condition would require a right response to the same stimulus. Such tasks demonstrating that certain mappings of stimuli to responses lead to faster response times and fewer errors than others are known as (stimulus-response) compatibility tasks and the observed performance differences as compatibility effects (for an overview, see Proctor & Vu, 2006).

Such a compatibility effect is also present when the ipsi-/contralateral decision is based on a different perspective. Müsseler, Ruhland, and Böffel (2019) asked participants to solve a compatibility task from an avatar’s point of view. Depending on the color of a stimulus, their response had to be ipsilateral or contralateral to the stimulus, as seen from the avatar’s point of view. Müsseler et al. (2019) observed a compatibility effect from the avatar’s perspective, even in trials that would otherwise be incompatible from the participant’s own point of view. Throughout this article, we refer to this task as the *avatar compatibility task*. In the present study, we address the role of automatic response activation for this task.

The concept of automatic response activation has been part of cognitive psychology for decades and is featured in prominent models of perception and action, for instance in the dimensional overlap model (DOM) by Kornblum and colleagues (Kornblum, Hasbroucq, & Osman, 1990; cf. also Hedge & Marsh, 1975; Wallace, 1971). This model is frequently used to explain compatibility effects by postulating that the presentation of a stimulus elicits activation in two routes of information processing: One route of automatic response activation of the spatially corresponding response and another route that includes the mapping rules to retrieve the correct response. When both routes lead to the same response, facilitation and improved performance are observed. When both diverge, costs occur. If we apply this theoretical framework to stimulus-response compatibility tasks, it stands to reason that the observed compatibility effect is caused by an automatic activation of the response ipsilateral to the presented stimulus. If the stimulus position is task-irrelevant, such tasks are known as Simon tasks and interestingly the compatibility effect is still observed (Simon & Rudell, 1967).

The DOM can also be used to explain the results of other well-known paradigms in cognitive psychology such as the Stroop task (Stroop, 1935), where the DOM assumes that the task-irrelevant meaning of a color word automatically activates the naming of that word. Another example is the Eriksen-flanker task, where task-irrelevant distractors can cause the automatic activation of a response that is inconsistent with the required one (Eriksen & Eriksen, 1974). The common basis of these tasks is a conflict in information processing, and they are therefore often summarized as conflict tasks. Solving these conflicts has been associated with cognitive control (Botvinick, Braver, Barch, Carter, & Cohen, 2001), which is required to suppress the execution of the automatically activated response (Kim, Lee, & Cho, 2015).

The role of automatic response activation for SR compatibility effects has been investigated using Simon tasks with crossed hands in which compatibility is generally dependent on the location of the response and not (or only to a lesser degree) of the effector (Anzola, Bertoloni, Buchtel, & Rizzolatti, 1977; Riggio, de Gonzaga Gawryszewski, & Umilta, 1986). Anzola et al. (1977) concluded that the advantage of ipsi- over contralateral responses is a result of “elementary anatomical connectivity” and these results have often been interpreted as evidence against automatic response activation. Wascher et al. (2001) identify *translation theories* (cf. Hasbroucq, Guiard, & Ottomani, 1990; Hasbroucq & Guiard, 1991) as the other key contender for the explanation of compatibility. Here, the core assumption is that stimuli and responses are both organized in codes and that correspondence between codes accelerates responses because it facilitates the matching process during response selection. Hasbroucq et al. (1990), for example, argue that in the case of a conceptual match between stimulus and response, response selection follows an algorithm-like rule implementation if the mapping allows identifying a systematic relationship between stimuli and their responses. The idea that stimuli and response information are organized in codes is featured in several theories of human perception and action and not limited to translation theories. The principle of common coding (Prinz, 1992, 1997), for example, states that both action and perception share a common representation instead of relying on two distinct coding systems. This is a core principle in modern theories of perception and action, for example in the *Theory of Event Coding (TEC*; Hommel, 2009; Hommel, Müsseler, Aschersleben, & Prinz, 2001; cf. also Müsseler, 1999). This shared nature of codes implies that every activation caused by perception is automatically also activation of a code useable in action planning.

There are several experiments that produced results that have been interpreted as evidence for automatic response activation in compatibility tasks. One approach uses electroencephalography (EEG) and the so-called *lateralized readiness potential (LRP)*, a contralateral potential that precedes voluntary actions (de Jong, Wierda, Mulder, & Mulder, 1988; Deecke, Grözinger, & Kornhuber, 1976; Kutas & Donchin, 1974). Sommer, Leuthold, and Hermanutz (1993) measured an LRP based on the task-irrelevant stimulus position in a Simon task and interpreted it as evidence for automatic response activation. Eimer (1995) used arrows as cues in a compatibility task and observed an LRP that provided evidence in favor of an automatic response activation based on the direction of the arrow cue. The LRP can also be observed in so-called choice-by-location tasks, where the stimulus position is the relevant feature for response selection (van der Lubbe, Jaśkowski, Wauschkuhn, & Verleger, 2001).

Additionally, the time course of compatibility effects can be used to gain information about automatic processes. This approach offers deeper insights into the underlying mechanisms, because the reaction time distribution can reveal certain effects of a manipulation that do not influence overall mean reaction times (Heathcote, Popiel, & Mewhort, 1991). De Jong et al. (1994) performed a distributional analysis of the Simon effect and identified two components of automaticity: A conditional component that appears to be independent of reaction time and related to the translation of stimulus information into the correct response code and an unconditional component that is caused by the priming of spatially corresponding responses and independent of the task-mapping. This unconditional component leads to a larger Simon effect in short reaction times but a decreased Simon effect with increased reaction times. A reaction time distribution with a decreased Simon effect for increased response times is commonly observed in the classical Simon task with left and right stimuli, and is generally explained with a decay or inhibition of the automatic response activation over time. In even slower reaction times, a reversed Simon effect has been demonstrated. Hedge and Marsh (1975) have argued that a reversal of the Simon effect is the result of a strategy in which participants follow an identity rule, but reverse the outcome in a second step. This concept is also considered by the model of De Jong et al. (1994). Zhang and Kornblum (1997) pointed out that the observed decrease of the Simon effect over time also reflects the properties of the two underlying reaction time distributions, and argued that some of the experiments violate the necessary assumptions to draw the aforementioned conclusions. Interestingly, the time-dependency of spatial compatibility effects is not universal. Different compatibility tasks, for example the vertical Simon task or Simon tasks that use centrally presented stimuli, as well as compatibility tasks with relevant stimulus locations often show a different pattern (for an overview, see Proctor, Miles, & Baroni, 2011).

Even though compatibility effects are overall reliable, they can be influenced by additional manipulations, for example, regarding intention (Hommel, 1993a), reference frames (Böffel & Müsseler, 2019b, 2019a, 2020; Hommel & Lippa, 1995; Müsseler et al. 2019), the frequency of compatible and incompatible trials (Hommel, 1994), or instruction (Böffel & Müsseler, 2018; Heister & Schroeder-Heister, 1994). In the present study, we examine whether one of the central claims of the dimensional overlap model, the automatic activation of the spatially corresponding response, holds true in the context of the avatar-compatibility task in which compatibility is manipulated by the presentation of an avatar. In a series of experiments, we presented evidence that compatibility effects can be influenced by presenting an avatar next to the stimulus set (Böffel & Müsseler, 2019b, 2019a, 2020; Müsseler et al. 2019) . This avatar provided an alternative frame of reference and the results showed that the coding of the stimulus position was based on this reference frame. The result was a compatibility effect from the avatar’s point of view that supports the concept of visual perspective taking (cf. Freundlieb, Kovács, & Sebanz, 2016).

In the present study we use a similar task to Müsseler et al. (2019), where participants performed ipsilateral (on the same side) or contralateral (on the opposite side) responses to a target, seen from the avatar’s perspective. Additionally, we vary stimulus-onset asynchrony (SOA) to either allow or prevent automatic response activation caused by the stimulus onset by presenting the imperative target first and the avatar (the frame of reference) second. This delay between target and avatar presentation exploits the fleeting nature of spatial codes. Once the participants observe the target, a stimulus code is formed that includes its location. We know from past studies that this stimulus code’s influence quickly diminishes over time (De Jong et al. 1994; Hommel, 1993b). This could be a result of either active suppression (Ridderinkhof, 2002) or spontaneous decay (Hommel, 1994). The delay forces the participants to postpone the selection of the correct response until the avatar appears on the screen, as the avatar is needed to identify the correct response. Even if the target onset elicits the automatic activation of any kind of response (e.g., a random response), the participants must suppress its execution until they have taken the avatar’s perspective into account. Most importantly, selecting the correct response is only possible after the perspective of the avatar is revealed. Any influence of automatic response activation should have vanished at this point, due to its volatility.

The idea of postponing response execution has been used before. Simon, Acosta, Mewaldt, and Speidel (1976), for instance, asked their participants to postpone the response execution in a compatibility task until a go stimulus was presented with a delay between 0 and 350 ms. Their results showed that a 250-ms delay is sufficient to eliminate the compatibility effect. Other studies estimated that the decay of the involved codes is slower. Roswarski and Proctor (1996) reported a time frame of approximately 700 ms, but the usual estimates range from 300 to 400 ms (cf. Zhang & Johnson, 2004). One explanation for the time dependency of the Simon effect is the decay of response-code activation over time (Hommel, 1994). Overall, this decay of automatic response activation seems rather fast (Eimer, Hommel, & Prinz, 1995) and we chose a delay of 750 ms in the present study to make sure it is completed at the time of response selection. If the automatic activation of the corresponding response is the driving factor behind the avatar-based compatibility effect, it should be significantly diminished if the automatic response activation is decayed.

## Experiment 1

**Fig. 1:**
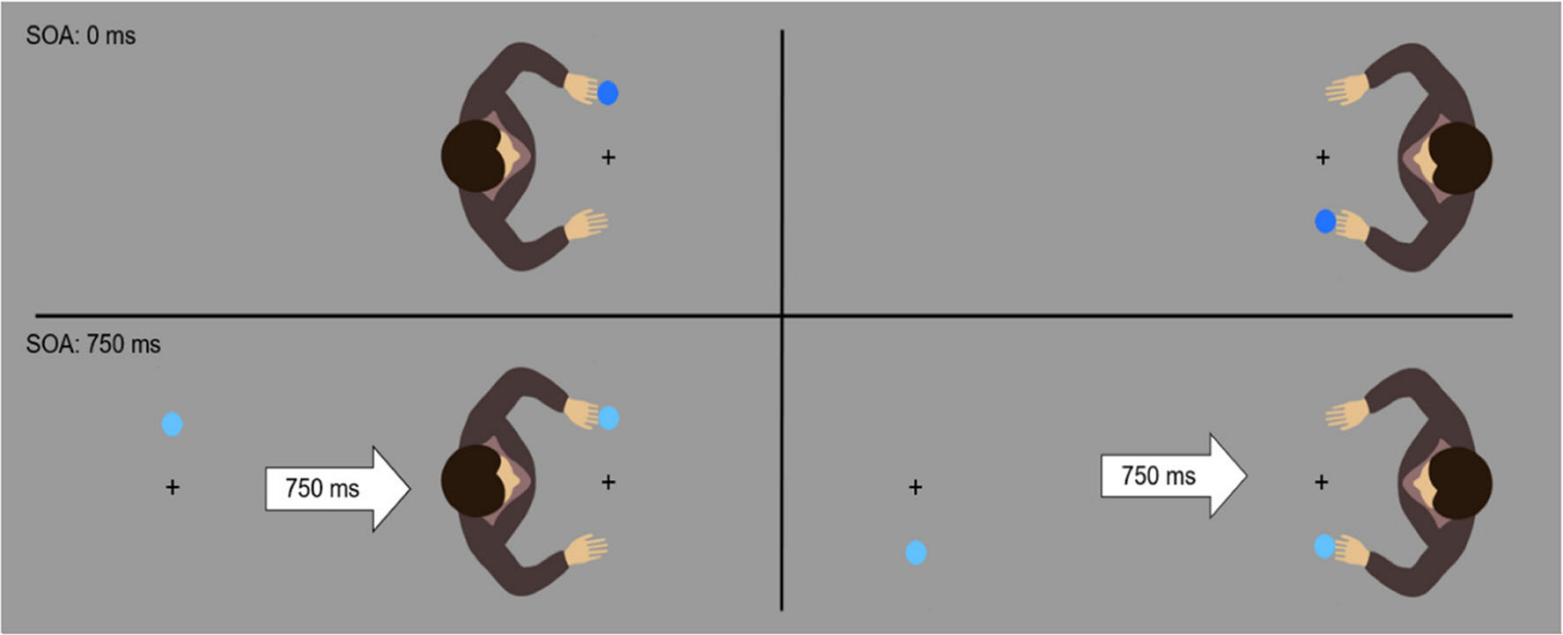
Avatars and setup of Experiment 1 with example conditions. **Left:** Avatar presented on the left. **Right:** Avatar presented on the right. **Top:** Without stimulus-onset asynchrony (SOA). **Bottom:** With an SOA of 750 ms. SOA, target position, target color, and avatar position were randomized within each block on a trial-to-trial basis

In the first experiment, we asked participants to perform ipsi- or contralateral responses to visual targets from the perspective of an avatar. The avatar is therefore task-relevant and cannot be ignored. The targets and the avatar were rotated by 90° either clockwise or counterclockwise to the perspective of the participants so that upper and lower position from the participants' point of view corresponded to left or right positions from the avatar’s perspective (Fig. 1).

In one condition, the avatar and the target appeared simultaneously. In these trials, the participants have both perspective and target information at their disposal at once and therefore automatic response activation in the relevant dimension is theoretically possible with the presentation of the target, if the target is immediately regarded within the reference frame provided by the avatar. If the target appears to the right of an avatar, automatic response activation should facilitate a right response. If the target appears on the avatar’s left, a left response should be facilitated instead (Fig. 1, top). In the delayed condition, the target presentation will precede the avatar presentation and the participants must wait for the avatar to appear to select the correct response (Fig. 1, bottom). This means that if automatic response activation follows the target position, it would be on the wrong dimension, on an up-down axis, instead of the left-right axis. Furthermore, a stimulus-response asynchrony (SOA) of 750 ms ensures that any automatic response activation will have decayed when the avatar finally appears. This task has some similarities to the one used in a compatibility task by Shaffer (1965), who also presented the target first, followed by a cue that contained the mapping information and observed an elimination of the compatibility effect. The avatar in our task fulfills a role similar to Shaffer’s cue, but there are some important differences. Instead of conveying whether the response is ipsi- or contralateral, the avatar only specifies which of the two response alternatives are ipsi- or contralateral. The information about the mapping itself is contained within the target and its color. If automatic response activation is a relevant factor for the avatar-compatibility effect, the compatibility effects should be larger without the delay. If we assume that automatic response activation is the main mechanism behind these compatibility effects, the compatibility effects should vanish in the delay condition.

## Method

### Participants

A total of 24 participants (19 female), students from RWTH Aachen University, with a mean age of 22.3 years (*SD* = 3.4) took part in this experiment. All participants reported normal or corrected-to-normal vision and gave informed consent. The sample size was determined based on an estimate of effect sizes typically observed in similar paradigms using G*Power (Faul, Erdfelder, Lang, & Buchner, 2007). If we take the effect size of the avatar’s influence reported by Müsseler et al. (2019) of 휂_푝_^2^ = .357, we would achieve a sufficient Power of (1- β) = 93% with this sample size.

### Apparatus and stimuli

The experiment was run in Matlab using the Psychtoolbox Extension v3.0 (Brainard, 1997; Pelli, 1997). The stimuli were presented on a 22-in. CRT monitor with a resolution of 1,024 × 768 pixels at a refresh-rate of 100 Hz. The participants were seated approximately 60 cm in front of the monitor and performed key presses on a horizontally oriented set of response keys using their index fingers. Target stimuli were dark blue (RGB: 36, 115, 254) and light blue (RGB: 98, 193, 254) discs with a diameter of 25 pixels, presented above or below a central fixation cross and in front of an avatar (240 × 190 pixels) on a gray (RGB: 155, 155, 155) background (Fig. 1).

### Procedure

We instructed the participant to perform either an ipsilateral or a contralateral response to the targets from the avatar’s perspective by pressing right or left keys on the keypad in front of them. The targets were presented either above or below a central fixation point and in front of an avatar that was facing the target. The target color (light or dark blue) determined the required mapping (ipsi- or contralateral) and the assignment of mapping to target color was counterbalanced between participants. If, for example, an ipsilateral response was required by the mapping and the stimulus appeared to the avatar’s right, participants had to perform a right key press. If the color called for a contralateral response, the participants had to perform a left key press instead. Depending on the avatar’s position, a stimulus presented below the fixation point could be on the avatar’s left or right side. This way, participants had to take the avatar’s perspective into account to select the correct response and postpone their response selection in the delayed condition until the avatar appeared.

Every participant completed one practice block with 48 trials followed by 14 experimental blocks with each block including three repetitions of each combination of target position (top and bottom), response position (left and right), avatar position (left and right), and SOA (0 and 750 ms) for a total of 672 trials with randomized order within each block. In the 750-ms SOA condition, each trial started with the presentation of the fixation cross and the target, followed 750 ms later by the presentation of the avatar either on the left or right side of the screen (Fig. 1). In the 0-ms SOA conditions, the target and avatar were presented simultaneously instead. The next trial started 1,500 ms after the participant’s response. Wrong responses and responses faster than 100 ms or slower than 1,500 ms were followed by an error sound each to discourage anticipatory responses and timeouts. Each error sound prolonged the pause between trials by an additional 1,500 ms. The participants took about 40 min to complete the experiment.

### Design

The experimental conditions formed a 2 × 2 × 2 design with the factors *SOA* (0 vs. 750 ms), *avatar position* (left vs. right), and *compatibility* (compatible vs. incompatible; from the avatar’s perspective) and repeated measures on all factors.

## Results

The first 48 trials were excluded from the data analysis as practice trials. We removed outliers (3.8 % of all trials) using the Tukey criterion (1.5 × interquartile range above the third or below the first quartile corresponding to each each mean that entered the analysis) and false responses (5.2 % of all trials) from the reaction time analysis. Mean reaction times and percentage errors (defined as % false responses of all responses) were analyzed separately using a 2 × 2 × 2 ANOVA with repeated measures on all factors.

### Reaction times

**Fig. 2:**
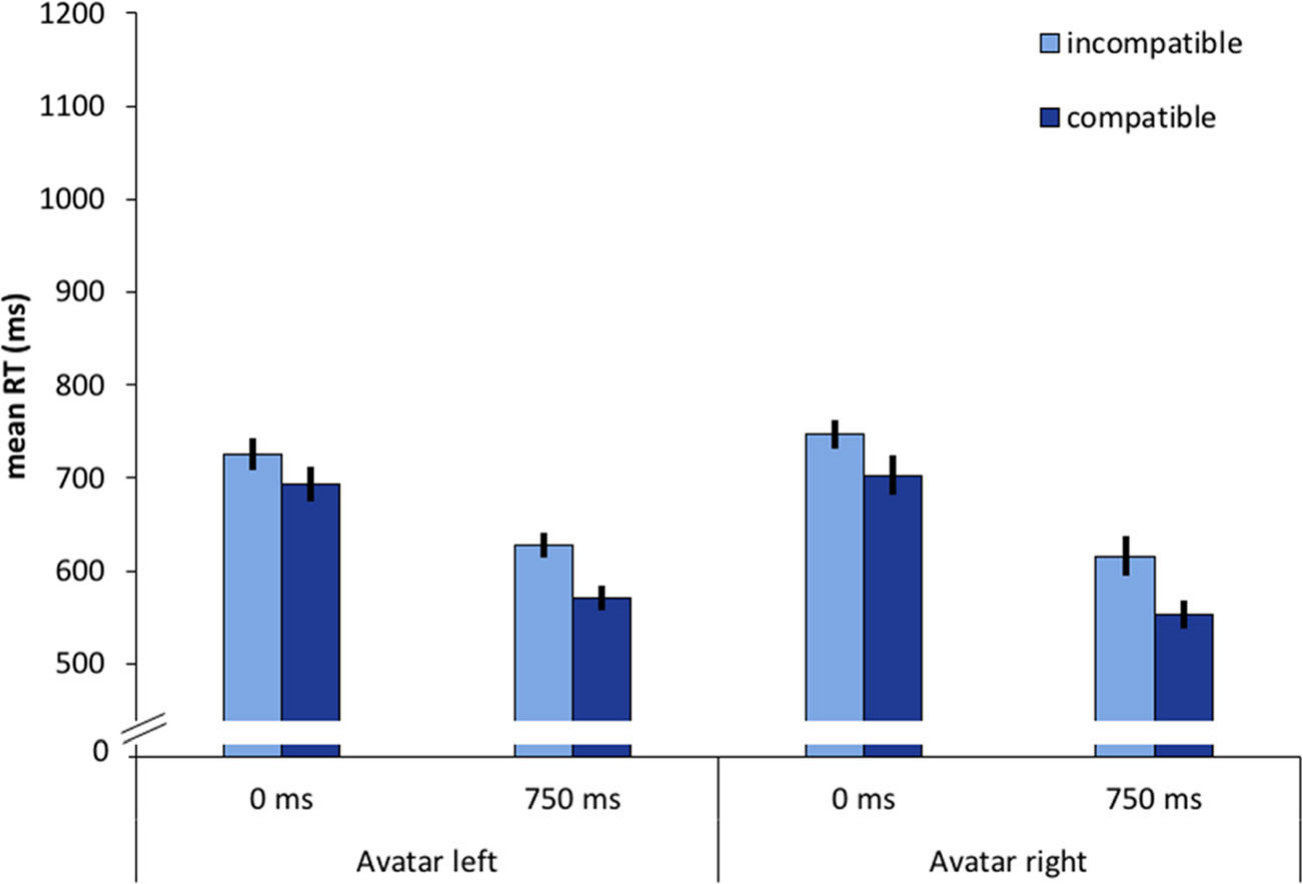
Mean reaction times (RT) as a function of avatar position (left vs. right), stimulus-onset asynchrony (0 ms vs. 750 ms) and compatibility, defined from the avatar’s perspective (compatible vs. incompatible). Error bars represent 95% within-subject confidence intervals (Morey, 2008)

We observed a significant main effect of compatibility*, F*(1, 23) = 32.49, *p* < .001, 휂_푝_^2^ = .586. Conditions that were compatible from the avatar’s point of view were associated with 49-ms faster responses compared to incompatible conditions. We also obtained a significant main effect of *SOA, F*(1, 23) = 203.41, *p* < .001, 휂_푝_^2^ = .898. The 750-ms SOA led to 125 ms faster responses compared to the 0-ms SOA conditions. We further observed a significant interaction of SOA and avatar-compatibility with *F*(1, 23) = 6.26, *p* = .020, 휂_푝_^2^ = .214, and the avatar-compatibility effect was larger in the 750-ms condition compared to the 0-ms condition. Additionally, the interaction of SOA and avatar position reached significance with *F*(1, 23) = 15.74, *p* = .001, 휂_푝_^2^ = .406. In the 0-ms condition, reactions were 16 ms faster when the avatar was on the left, in the 750-ms condition reactions were 15 ms faster with the avatar on the right. Mean reaction times are shown in Fig. 2.

### Percentage errors

**Fig. 3:**
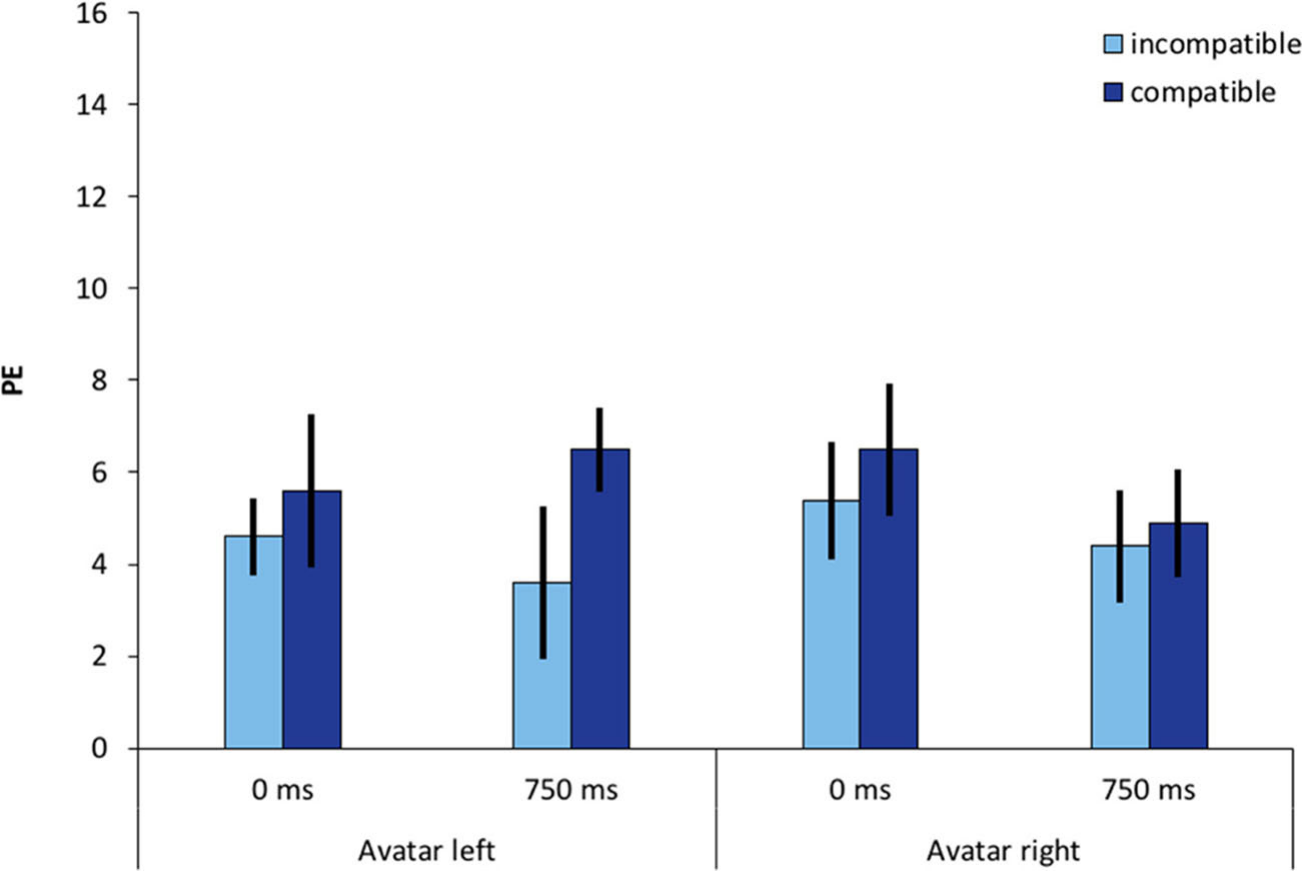
Percentage errors (PE) as a function of avatar position (left vs. right), stimulus-onset asynchrony (0 ms vs. 750 ms), and compatibility, defined from the avatar’s perspective (compatible vs. incompatible). Error bars represent 95% within-subject confidence intervals (Morey, 2008)

We observed a significant main effect of compatibility on percentage errors with *F*(1, 23) = 4.75, *p* = .04, 휂_푝_^2^ = .171 with 1.4 %-points lower error rates for incompatible conditions compared to compatible ones. We discuss this finding below. The two-way interaction of compatibility and SOA was not significant with *F*(1, 23) = 1.13, *p* = .30, 휂_푝_^2^ = .05. No other significant effects were observed. Mean percentage errors are shown in Fig. 3.

### Post hoc Bayes t-test

Since the direction of the observed main effect in compatibility and of the SOA–compatibility interaction was opposite, we were faced with the problem of differences in speed-accuracy tradeoffs between the conditions. The main goal of this study was to test the hypothesis that a larger compatibility effect is observed in the no-delay condition. Even though the significant effect in reaction times is greater than the non-significant effect in errors, the reaction-time data goes against the initial hypothesis, while the direction of the percentage error effect is aligned with the hypothesis. While our analysis yields evidence for the opposite effect in response times, the non-significant result in percentage error is hard to interpret within the framework of null-hypothesis significance testing, since the analyses do not allow us to quantify the evidence against the hypothesis. We therefore decided to run directed, post hoc Bayes t-tests of the compatibility effects in reaction times and percentage errors between each SOA condition with JASP (JASP Team, 2019), aligned with the initial hypothesis (greater compatibility effect in the 0-ms SOA condition). We used the default Cauchy prior width of *r* = .707. The RT analysis yielded strong evidence against the hypothesis (BF_+0_ = 0.0705), while the analysis of percentage errors yielded anecdotal evidence against the hypothesis (BF_+0_ = 0.596).

### Reaction-time distribution

**Fig. 4:**
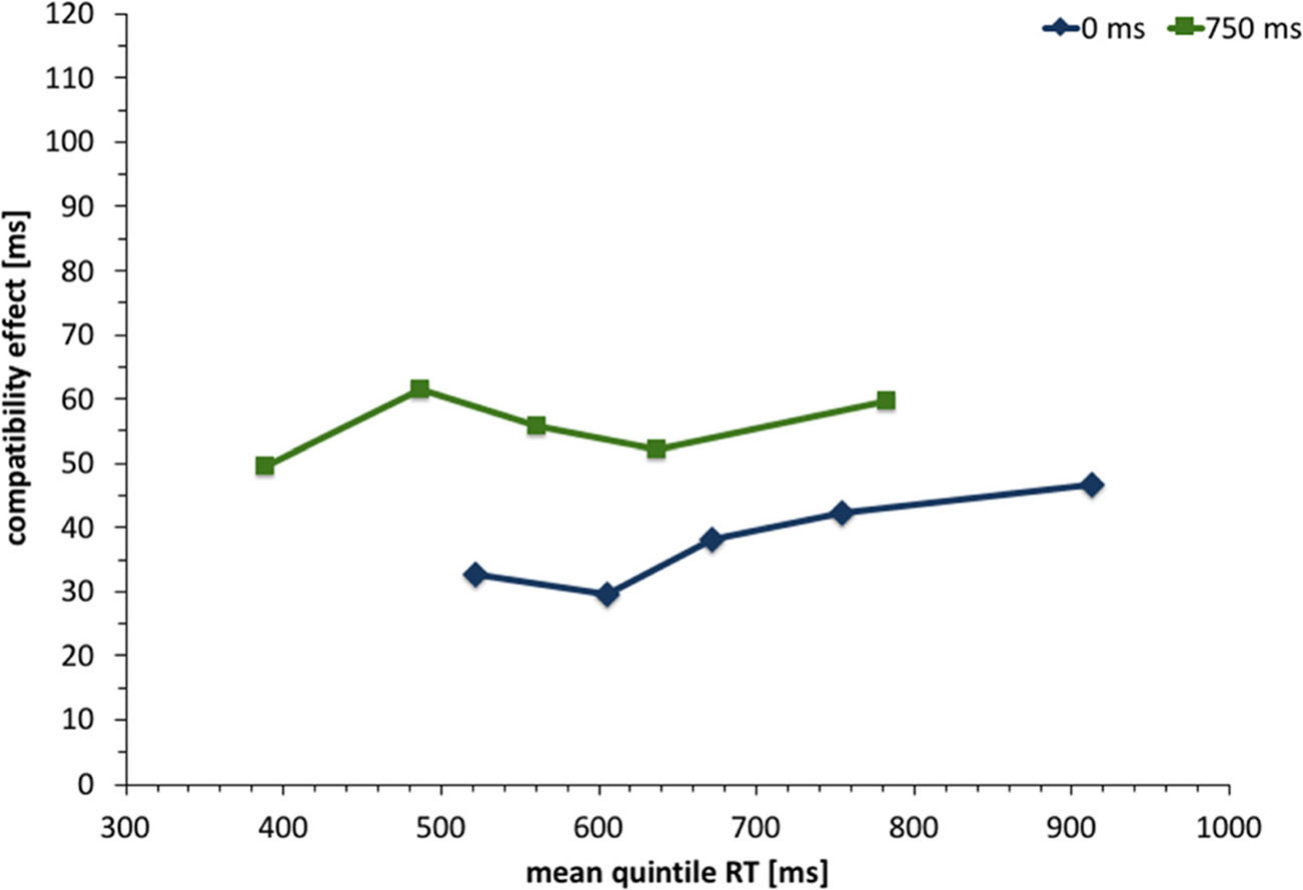
Compatibility effect (incompatible – compatible condition) in Experiment 1 as a function of reaction-time bin (quintile). Compatibility was defined from the avatar’s point of view. For instance, conditions in which the stimulus was presented to the avatar’s right were compatible with a right key press and incompatible with a left key press

To examine potential changes of the compatibility effect with faster or slower reaction times, a distributional analysis (Fig. 4) was conducted by calculating reaction-time bins (quintiles) for each participant and condition. We conducted a repeated-measures ANOVA with Greenhouse-Geisser correction and the factors *SOA*, *compatibility*, and *reaction-time bin*, to examine whether the compatibility effect changed with reaction time. However, no significant interactions including reaction-time bin were observed and the interaction of compatibility and reaction-time bin reached *F*(1.14, 26.23) = 0.63, *p* = .64, 휂_푝_^2^ = .027.

## Discussion

In reaction times we were able to clearly replicate the avatar-based compatibility effect with task-relevant avatars reported by Müsseler et al. (2019). The participants again showed that spatially corresponding conditions lead to faster response times compared to non-corresponding conditions, indicating that the targets were coded from the avatar’s point of view as right and left, instead of up or down. The result is a compatibility effect that favors compatible over incompatible responses from the avatar’s perspective.

However, the effect is reversed in percentage errors with worse performance in the compatible conditions indicating a speed-accuracy tradeoff (Wickelgren, 1977). But because we observed only very few errors in general and because the effect in percentage errors is much smaller compared to the effect in mean reaction times, we can conclude that a performance advantage is observed for the compatible conditions.

We further found a substantial effect of SOA on mean reaction times. We think that the facilitation caused by the SOA shows that the participants were able to prepare their response during the SOA by simplifying the response selection. If the target, for example, demanded a compatible response, the participants could disregard the incompatible responses, reducing the task complexity. Why this is an advantage is not obvious at first glance, because the participants still must select between left and right responses after the avatar presentation, leaving the number of responses to select from unchanged. However, since the mapping rule is known before the avatar appears, the task is very similar to a compatibility task that has only compatible or incompatible responses varied in blocks. In both tasks, the participants know the mapping rule in advance and can use this information for response preparation.

The observed significant interaction of SOA and avatar position could be a result of attentional asymmetries that follow reading direction (cf. Rinaldi, Di Luca, Henik, & Girelli, 2014). In the 0-ms SOA condition, participants might favor the left avatar position over the right one, as this allows scanning the scene following the reading direction. The opposite is true for the 750-ms SOA condition, where attention lies on the target first and has to be shifted to the avatar. In this case, the shift follows the reading direction if the avatar is on the right.

Most important for our present research question, the SOA’s influence on compatibility in mean reaction times was significant, but the direction was reversed, compared to the prediction by the automatic response activation account. The 750-ms SOA did not reduce the compatibility effect, but instead increased it. These results provide evidence that automatic response activation is not responsible for the compatibility effect but, instead, a general advantage for compatible (ipsilateral) over incompatible (contralateral) responses.

One fact that can be interpreted as additional evidence against the role of automatic response activation can be found in the distributional analysis of reaction times. A decreased compatibility effect with increased reaction times has traditionally been interpreted as evidence for the decay of automatic response activation. However, in this experiment, we find no relevant difference in the reaction-time distribution of either SOA condition. This leads us to believe that the role of automatic activation is similar in both cases, i.e. likely irrelevant.

One additional point to consider is the role of the SOA manipulation overall. Since the 0-ms SOA condition appears to be more difficult and produces significantly higher overall reaction times compared to the 750-ms condition, this difference in overall mean reaction times alone could have caused differences in the reaction-time distribution between the 0- and 750-ms SOA conditions: The reaction-time distribution of the 0 ms condition was shifted to the right when compared to the distribution of the 750-ms condition. If automatic response activation is a relevant factor in this paradigm, this shift could be problematic if the compatibility is unstable across different reaction times (e.g., larger in smaller reaction). The automatic activation would have more time to decay in the 0-ms condition, based on the overall larger reaction times alone. However, the results show that the compatibility effect over time is indifferent to the change in overall mean reaction times, which makes it unlikely that the difference in mean reaction times between both SOAs is responsible for the difference in compatibility effects.

One major caveat of this experiment lies in the orthogonal nature of the task. Because the target and response positions varied on different dimensions, the dimensional overlap was only introduced after the targets were coded from the avatar’s perspective. It could therefore be argued that even if the target presentation leads to an automatic activation, this activation would be expected on a vertical up-down axis, instead of in the right-left dimension. A stimulus presented at the top could, for example, prime an upper response. However, such an upper response is not part of the response set used in our experiment, and any possibility of such an effect appears to be easily dismissible at first glance. Nevertheless, it is worth noting that the results of orthogonal compatibility tasks seem to counter this line of reasoning (cf. Bauer & Miller, 1982; Cho & Proctor, 2005; Nishimura & Yokosawa, 2006), and suggest that the possibility of an orthogonal influence should still be considered. It could, for example, be possible that a stimulus at the top automatically activates a right response, following the general direction of orthogonal compatibility effects. Despite that, we found no evidence for an orthogonal compatibility effect as such an effect would have resulted in a significant interaction of compatibility and avatar position. Because the orthogonal compatibility effect usually leads to an advantage of the right-up/left-down mapping compared to the reverse, this effect would have been aligned with the compatibility when the avatar is on the right, but opposite when the avatar is on the left. With the avatar on the right, a compatible SR-pair is also orthogonally compatible, while avatar-incompatible conditions are orthogonally incompatible. When the avatar is on the left, the opposite is true. Conditions that are compatible from the avatar’s point of view are orthogonally incompatible and vice versa. Therefore, any significant orthogonal compatibility effect would lead to a significant interaction of compatibility and avatar position. However, this was not found, and we can conclude that no orthogonal compatibility effect was present.

Another aspect to consider is that the orthogonal compatibility effects are relatively small and automatic response activation might be reduced compared to parallel tasks. It therefore seems worthwhile to change the task to allow for automatic response activation in the left-right dimension that would constitute an unmediated dimensional overlap with our response dimension.

## Experiment 2

**Fig. 5:**
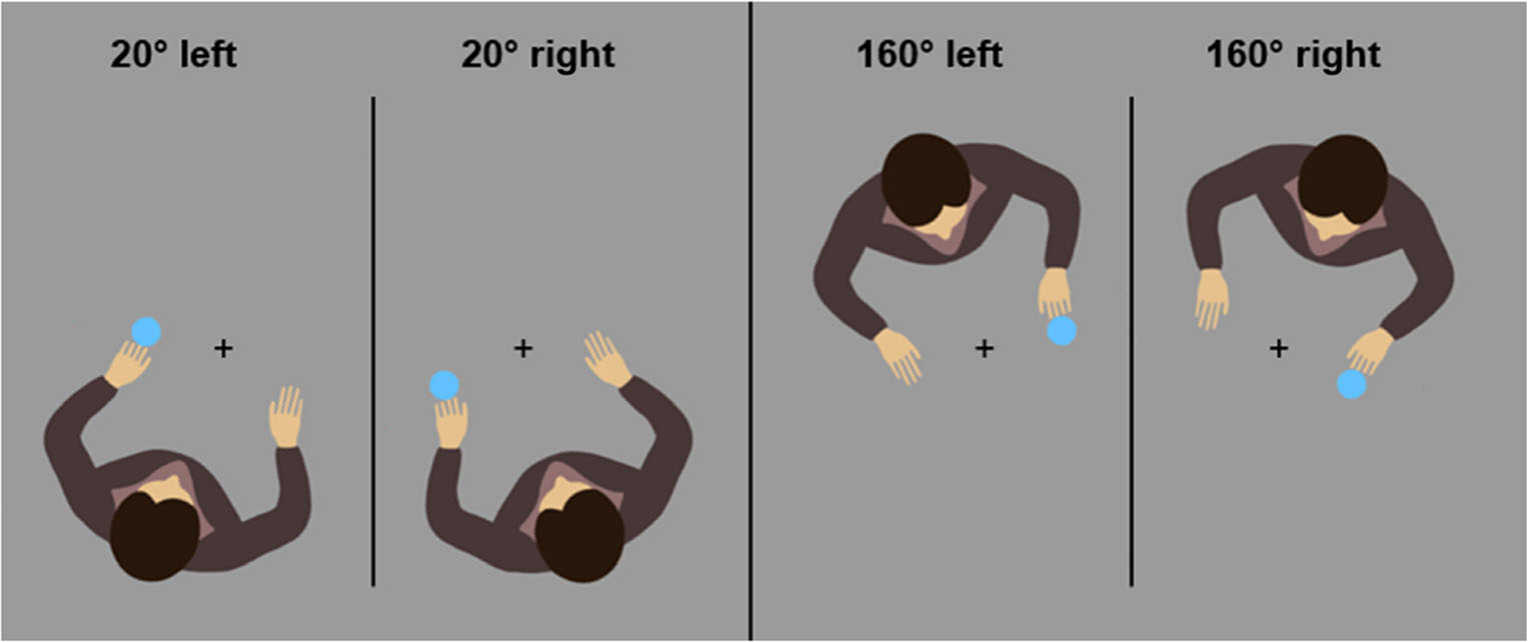
Avatar rotations used in Experiment 2. The target locations and the avatar were rotated around a centrally presented fixation cross by either 20° or 160° from the participant’s point of view, to the right and left. In the 20° conditions, target positions were the same on the left-right dimension from the avatar’s and the participant's point of view, here: left. In the 160° conditions, both perspectives conflicted, the stimulus is on the participant’s right, but on the avatar’s left

To test whether the results of Experiment 1 hold true for a compatibility task with lateralized target positions, we decided to repeat the experiment with a different setup. Instead of 90° rotation of targets and avatar, we now used 20° and 160° from the participant’s point of view so that the target position can be clearly identified as right or left (Fig. 5). This was not the case in the first experiment in which the stimuli could only be identified as left or right from the avatar’s point of view. But it is possible that an initial discriminability in the left-right dimension changes the results, because it could allow for automatic response activation on this dimension without the need to process the avatar’s perspective information.

## Method

### Participants

A total of 24 students from RWTH Aachen University (20 female) with a mean age of 21.3 years (*SD* = 2.3) participated in the experiment for course credit. All participants reported normal or corrected-to-normal vision and gave written informed consent.

### Apparatus, stimuli, and procedure

The general setup and procedure were the same as in Experiment 1, but the rotation of the avatar and targets were different. Instead of using left and right rotations of 90°, we now used left and right rotations of 20° and 160°. In the 20° rotation conditions, the spatial SR compatibility on the left-right dimension was the same, regardless of whether the target position was regarded from the participant’s perspective or the avatar’s perspective. In the 160° condition, combinations of stimuli to responses that were compatible from the participant’s point of view are now incompatible from the avatar’s perspective and vice versa. We chose 160° and 20° compared to 0° and 180° to avoid a situation in which the 180° rotation is interpreted as a mirrored instead of rotated. The participants performed a total of 23 blocks, including one of each combination of target position (right and left), response position (right and left), avatar position (20° right, 20° left, 160° right, 160° left) and SOA (0 ms and 750 ms) for a total of 688 trials. The first block was a practice block and discarded from the analysis. The participants took about 45 min to complete the experiment.

### Design

The design was similar to Experiment 1, with the exception that the factor *avatar position* had four manifestations (20° rotated to the left vs. 20° rotated to the right vs. 160° rotated to the left vs. 160° rotated to the right). Together with the factors *compatibility* from the avatar’s point of view and *SOA* the result is a 4 × 2 × 2 design with repeated measures*.*

## Results

Outlier identification was the same as in Experiment 1. We excluded 4.0 % response errors and 4.0% outliers from the reaction time analysis. Mean correct reaction times and percentage errors were analyzed separately using 4 × 2 × 2 repeated-measures ANOVA with Greenhouse-Geisser correction.

### Reaction times

**Fig. 6:**
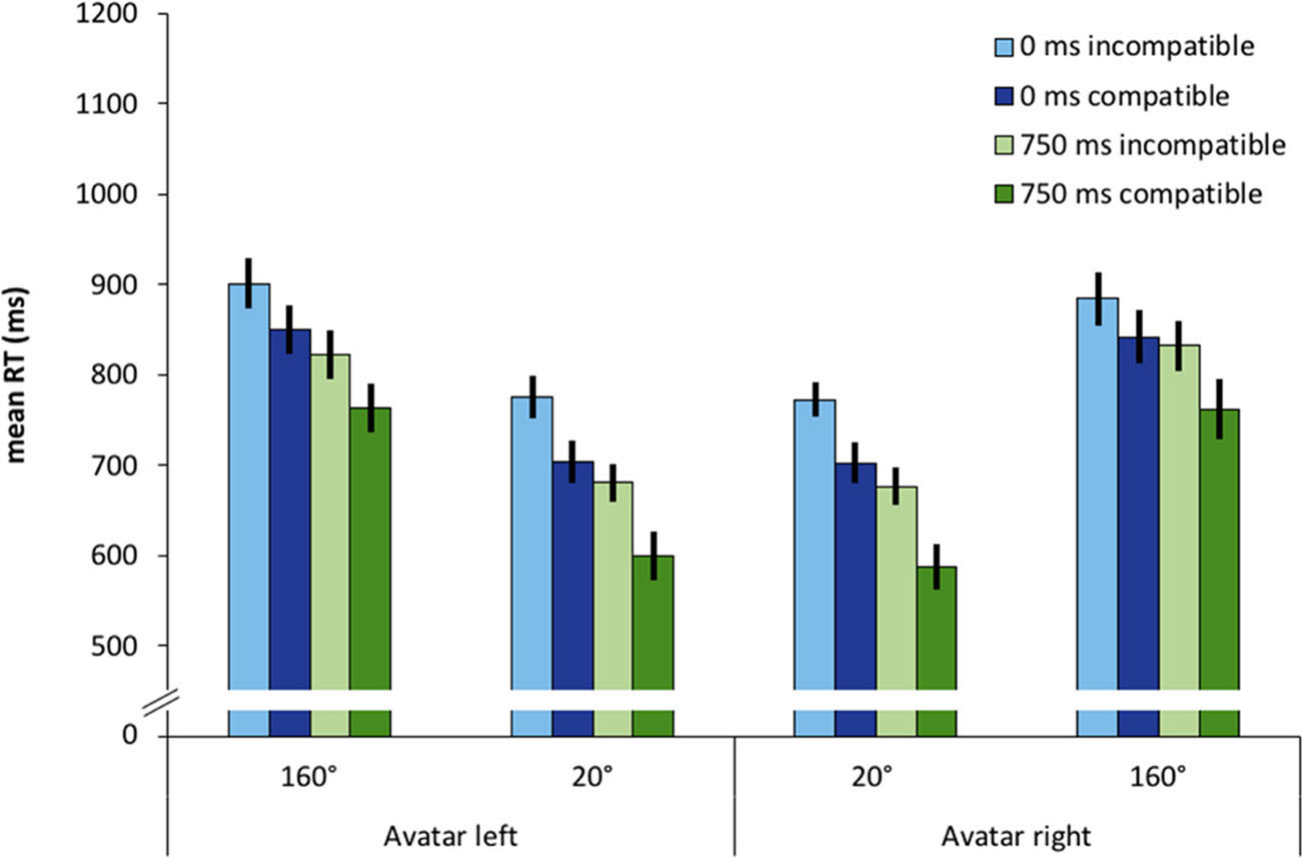
Mean reaction times (RT) as a function of avatar position (160° left, 20° left, 20° right, 160° right), stimulus-onset asynchrony (0 ms vs. 750 ms), and compatibility, defined from the avatar’s perspective (compatible vs. incompatible). Error bars represent 95% within-subject confidence intervals (Morey, 2008)

We observed a significant main effect of SOA with *F*(1, 23) = 242.38, *p* < .001, 휂_푝_^2^ = .913. Responses were 88 ms faster in the 750-ms condition. The main effect of compatibility was significant with *F*(1, 23) = 107.48, *p* < .001,휂_푝_^2^ = .824, and responses were 66 ms faster in the compatible conditions compared to incompatible ones. We further measured a significant main effect of avatar position with *F*(1.52, 34.98) = 102.18, *p* < .001, 휂_푝_^2^ = .816. Reactions were faster when the avatar was rotated by 20° compared to 160°, both for left and right rotations. The relevant interaction of compatibility and SOA reached significance with *F*(1, 23) = 6.047, *p* = .022, 휂_푝_^2^ = .208. The compatibility effect was overall larger in the 750-ms SOA condition. Mean reaction times per condition are shown in Fig. 6.

### Percentage errors

**Fig. 7:**
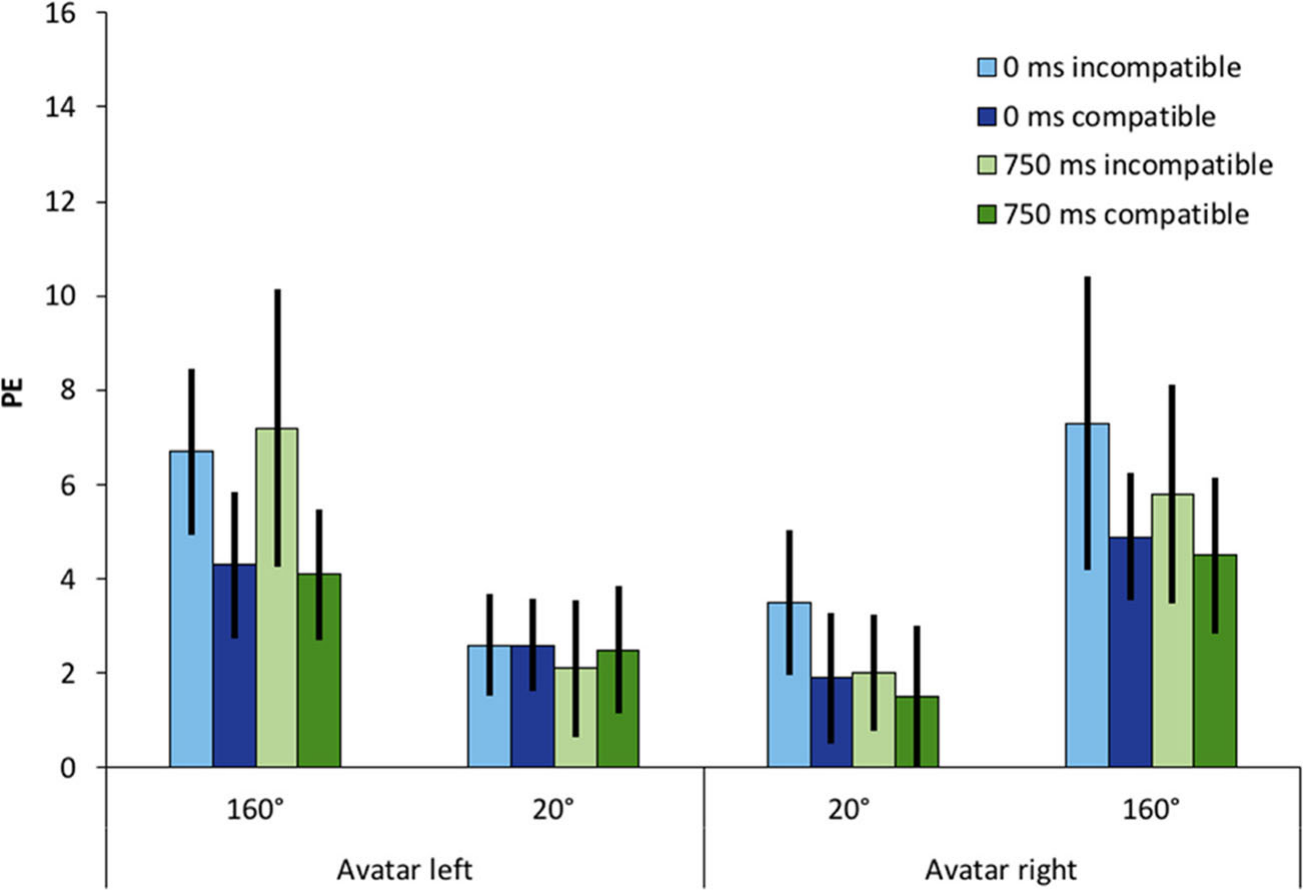
Percentage errors (PE) as a function of avatar position (160° left, 20° left, 20° right, 160° right), stimulus-onset asynchrony (0 ms vs. 750 ms), and compatibility, defined from the avatar’s perspective (compatible vs. incompatible). Error bars represent 95% within-subject CIs (Morey, 2008)

We observed a significant main effect of avatar position with *F*(1.26, 29.04) = 12.90, *p* < .001, 휂_푝_^2^ = .359. The relevant interaction of SOA and compatibility reached *F*(1, 23) = 0.58, *p* = .455, 휂_푝_^2^ = .024. Mean percentage errors per condition are shown in Fig. 7.

### Post hoc Bayes t-test

Similar to Experiment 1, the interactions of compatibility and SOA are opposite in reaction times and percentage errors. We again ran directed, post hoc Bayes t-tests of the compatibility effects in reaction times and percentage errors between each SOA condition with JASP (JASP Team, 2019), testing the initial hypothesis (greater compatibility effect in the 0-ms SOA condition) using the default Cauchy prior width of *r* = .707. Like Experiment 1, the reaction-time analysis yielded strong evidence against the hypothesis with BF_+0_ = 0.0713, while the analysis of percentage errors yielded anecdotal evidence against the hypothesis with BF_+0_ = 0.425.

### Reaction-time distribution

**Fig. 8:**
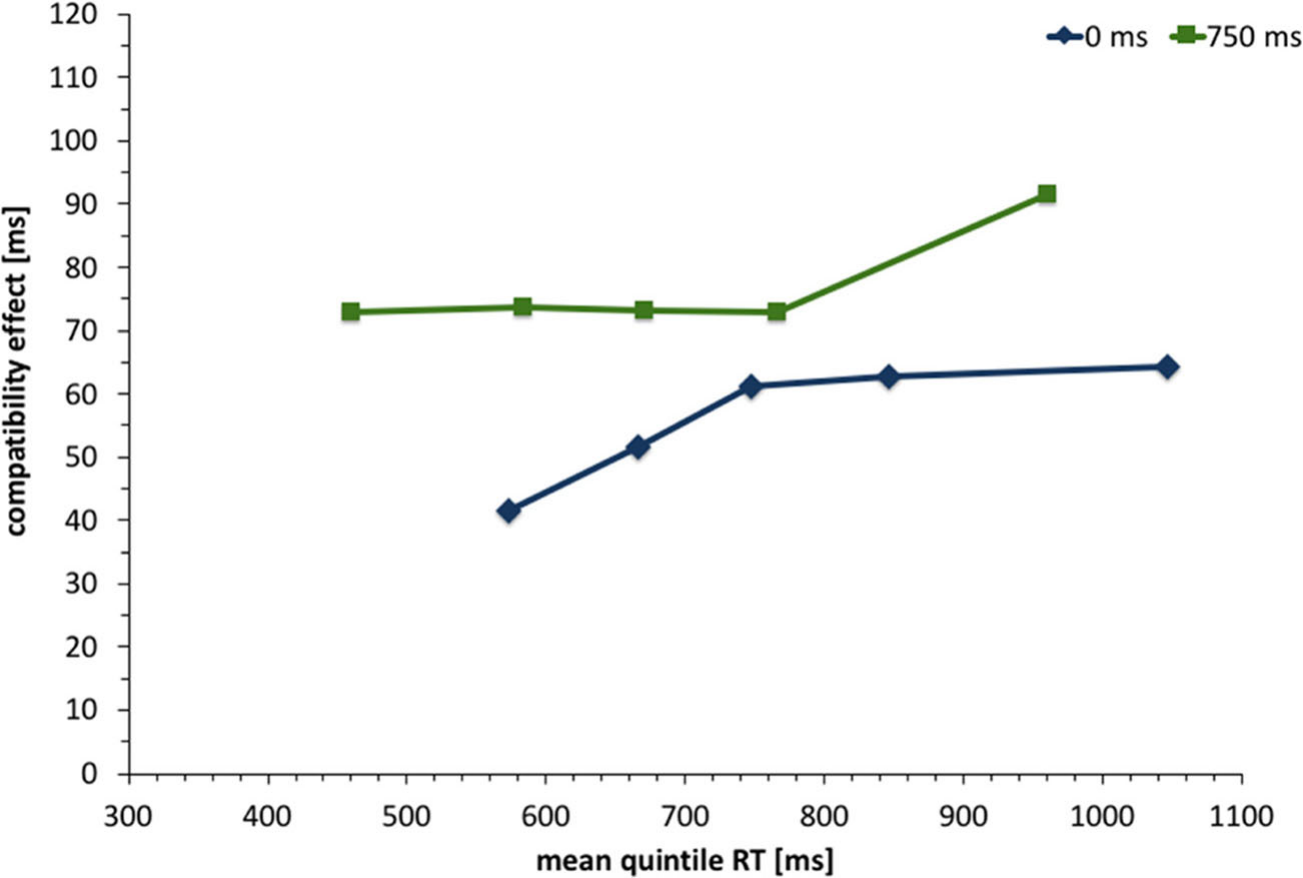
Compatibility effect (incompatible – compatible; defined from the avatar’s point of view) in Experiment 2 as a function of reaction time (RT) bin (quintile). Compatibility was defined from the avatar’s point of view, for example, conditions in which the stimulus was presented to the avatar’s right were compatible with a right key press and incompatible with a left key press

The distributional analysis (Fig. 8) revealed a significant interaction of reaction-time bin and SOA with *F*(1.83, 42.084) = 8.797, *p* = .001,휂_푝_^2^ = .277. With a larger influence of SOA in faster reactions compared to slower reactions. No other new significant interactions including reaction-time bin were observed and the interaction of compatibility and reaction-time bin reached *F*(1.578, 36.294) = 1.828, *p* = .181, 휂_푝_^2^ = .074. All analyses were Greenhouse-Geisser corrected.

## Discussion

The results of Experiment 2 clarify the results of Experiment 1. We replicated the substantial effects of SOA and compatibility from the avatar’s perspective. The interaction of SOA and compatibility was again significant with numerically larger compatibility effects in the 750-ms delay condition. This stands in contrast to the prediction of the automatic response activation account. Furthermore, the reaction-time distribution analysis revealed no significant interaction involving reaction-time bins and compatibility, indicating that the compatibility effect is relatively stable across reaction times. Most importantly, the pattern is again different from the negative slope, usually associated with the decay of the automatic response activation. The results support the conclusion that the automatic activation of responses that are spatially corresponding from the avatar’s perspective is not responsible for the compatibility effect from the avatar’s point of view. This is even the case in this set-up where the automatic activation would occur in the relevant left-right dimension and is in 50% of the cases opposite to the compatibility effect from the avatar’s perspective.

## General discussion

The goal of this study was to determine whether the automatic activation of compatible responses from the avatar’s point of view is the driving factor for the avatar-based compatibility effect. Models of compatibility such as the dimensional overlap model (Kornblum et al. 1990) propose the existence of an automatic route in which a stimulus and its features directly activate a corresponding response. We used a manipulation that would eliminate any influence of automatic response activation by delaying the response selection until this activation is decayed. Contrary to the prediction, we observed no reduction of the avatar-based compatibility effects in the delay condition; if anything, the compatibility effect appears to be more pronounced. However, this opposing effect in reaction times was accompanied by a reversed pattern in the error rates of Experiment 1, which could indicate a speed-accuracy tradeoff (Wickelgren, 1977). Using post hoc Bayes t-tests to quantify the evidence in favor of or against the automaticity hypothesis revealed anecdotal evidence against it in error rates and strong evidence against it in mean reaction times. The reaction time distributions of both experiments further supported the conclusion that automatic response activation was absent. A decreasing compatibility effect with increasing reaction times, attributed to a decay of the automatic response activation over time, was not observed in either SOA condition in either experiment.

Overall, we believe the results of all analyses combined constitute strong evidence against the influence of automatic response activation in this paradigm and casts additional doubt on the role of automatic response activation for compatibility, particularly in tasks with relevant target locations.

One important question remains: If the observed compatibility effect is not a result of automatic response activation, then where does it originate from? To reconcile the results of the present study with the dimensional overlap model (Kornblum et al. 1990), it can be argued that the experimental set-up did not lead to a dimensional overlap between stimuli and responses, thus not leading to automatic response activation. In such a case, the model could account for performance differences by ascribing faster response identification to ipsilateral conditions, as a result of a faster mapping-rule. But why is the ipsilateral mapping faster, even in the absence of automatic response activation? We favor the following alternative explanation, which is based on an earlier idea of Hedge and Marsh (1975; see Proctor et al. 2011): The incompatible, contralateral, or opposite response could be a derivative of the compatible, ipsilateral, or same response. The idea behind this is that the task is primarily approached by focusing on the same-side response, and if this response happens to be wrong, a second step is introduced that reverses the response proposed by the sameness-rule. Or to put it differently, the task is approached in a hierarchical manner, with same reaction always taking preference over opposite reaction. This could overall be beneficial if the time gained in compatible conditions outweighs the additional cost of the inversion step in incompatible trials. Other strategies might be used as well. We observed, for example, higher error rates in compatible compared to incompatible conditions in Experiment 1, which indicates a speed-accuracy tradeoff that was absent in Experiment 2. The most likely explanation for this difference between both experiments is that the task in Experiment 1 is overall easier. This idea is supported by the overall higher reaction times in Experiment 2 and likely a result of additional costs introduced by larger angular disparities between person and avatar. While the 90° rotation is rather easily bridged, the costs for the 160° rotation seem to outweigh the benefits of the 20° rotation relative to the 90° in Experiment 1 (cf. Janczyk, 2013). The participants might have adapted to this by favoring speed over accuracy in Experiment 1, but not in Experiment 2.

We can also apply the principle of common coding to the results, for example as described by the theory of event coding (Hommel, 2009; Hommel et al. 2001). Performing a left response shares feature codes with observing a left stimulus. Regardless of whether perception or action are responsible for the creation of the feature code “left,” both are functionally the same. The outcome of this feature-binding process has been labelled *event file* (Hommel, 1998). One advantage of compatible responses could be that the formation of the response code is facilitated because the needed feature code “left” is already activated and part of the relevant event file. While this activation of the shared event file is by definition automatic, it does not directly translate into the automatic activation of a specific response, since a vast amount of possible actions could share the same feature code. At the same time, other feature codes needed to perform the response might not be recruited yet. The response is not completely formed and therefore not automatically activated, even though some shared feature codes might be. The activation of certain feature codes might therefore be a necessary but not a sufficient condition for automatic response activation. One candidate for the regulation of automatic activation is the concept of *metacontrol* proposed by Hommel and Wiers (2017). Hommel and Wiers argue that the strict dichotomy of automaticity versus intentionality should be abandoned in favor of a “unitary approach” to action control. Metacontrol serves as a mechanism that determines to what degree automatic processes influence action control. In our experiments, assuming a metacontrol state that reduces the influence of automatic processes would be sensible since it not only prevents activating the wrong response in the 50% contralateral cases, but also a response on a wrong vertical axis in Experiment 1. Furthermore, it prevents automatic response activation caused by the avatar’s position itself.

Similarly, the role of cognitive control can be discussed. Cognitive control enables us to adapt to a specific situation by helping us focus on certain aspects, generally at the cost of flexibility (Botvinick et al. 2001). Most of the results regarding cognitive control and compatibility are based on conflict-monitoring tasks, where task-irrelevant features must be ignored, such as the position of a stimulus in a Simon task, or the flankers in an Eriksen flanker task. The system of cognitive control is flexible and able to quickly adapt to changes on a trial-by-trial basis. It is known, for example, that incompatible trials lead to an increase in cognitive control in the following trial and a reduced compatibility effect (Botvinick et al. 2001; Egner, 2007; Gratton, Coles, & Donchin, 1992). The task we used is different as the conflict is not caused by an irrelevant distractor, but by differences in the stimulus and response locations. However, our task still features compatible and incompatible trials. Because the participants knew the mapping in advance in the 750-ms delay condition, it is possible that this resulted in changes in cognitive control. Generally, in compatibility tasks, an increase in cognitive control is associated with reduced compatibility effects. However, it is important to note that this is a double-edged sword, as cognitive control not only reduces the additional performance costs of incompatible conditions, but also the potential performance benefits of compatible conditions. Interestingly, the situation is different if the participants know that they must perform a compatible response in advance. In this case, cognitive control could negatively influence overall performance, and reducing it should increase the compatibility effect, benefitting overall performance. On the contrary, if the participants know that the required response is incompatible, then cognitive control might be helpful to reduce the compatibility effect that would now reduce performance. Of course, this strategy cannot be applied if the mapping is not known in advance, explaining a potentially larger compatibility effect in the delayed condition.

## Conclusion

In sum, the results lend no evidence to an automatic-response activation account of the avatar-based compatibility effect. Instead, it seems likely that an overall advantage of ipsilateral responses compared to contralateral responses is the driving factor behind this effect, which is more accurately explained within frameworks of common coding and metacontrol. The effect might also reflect a hierarchical strategy during response selection that is independent of automatic response activation or modulated by cognitive control.

## Electronic supplementary material

(ZIP 7.64 kb)
